# Having female role models correlates with PhD students’ attitudes toward their own academic success

**DOI:** 10.1371/journal.pone.0255095

**Published:** 2021-08-18

**Authors:** Shauna N. Gillooly, Heidi Hardt, Amy Erica Smith

**Affiliations:** 1 Department of Political Science, University of California, Irvine, Irvine, California, United States of America; 2 Department of Political Science, Iowa State University, Ames, Iowa, United States of America; University of Westminster, UNITED KINGDOM

## Abstract

Research indicates that increasing diversity in doctoral programs can positively affect students’ academic success. However, little research examines students’ responses to female scholars’ representation. The two studies presented here examine how students’ exposure to female academic role models shapes students’ attitudes toward their own academic success (i.e. self-efficacy). Such attitudes are critical because they predict student retention rates. In our first study, we randomly exposed 297 Ph.D. students in one academic discipline to either a gender-diverse (i.e. 30% female authors) or non-diverse syllabus in research methods (i.e. 10% female authors). We examined the effect of the intervention on students’ perceived likelihood of succeeding in the hypothetical course. Contrary to expectations derived from the literature, we found that increasing women’s representation in syllabi did not affect female students’ self-efficacy. Rather, male students expressed lower self-efficacy when evaluating the more gender-diverse syllabus. We also found that students’ attitudes toward diversity in academia predicted their reactions more strongly than did their own gender: gender-diverse syllabi reduced self-efficacy among those students unsupportive of diversity. In our second study, we analyzed non-interventional survey questions to examine the relationship between female role models and long-term academic self-efficacy. Analysis was observational and thus did not assess causality. We found that students with more role models have higher academic self-efficacy, irrespective of student and role model gender. Nonetheless, results also suggested that some students actively seek female role models: namely, female students, and particularly those valuing diversity. Our results ultimately suggest that exposure to female role models relates in surprising ways to Ph.D. students’ self-efficacy. Having more female role models correlates with greater expectations of academic success among certain groups of students, but with diminished expectations of academic success among other groups.

## Introduction

Doctoral programs continue to struggle with the problem of retention, with about half of students leaving before completing their degrees [1, 2]. Women and students of color are even more likely than white and male students to leave [3, 4]. Whereas similar numbers of men and women enter graduate school, women depart at higher rates than men, even when the latter academically outperform the former [5]. This article focuses on one Ph.D. student attitude that is a key predictor of students’ retention: self-efficacy, or students’ perceptions of their own likelihood of achieving academic success [2, 6].

How does exposure to female scholars affect female and male students’ self-efficacy? Our project’s principle aim was to test the impact of an understudied structural characteristic of many Ph.D. programs–students’ low exposure to female academic role models–on self-efficacy. Past research has found that academic role models, and faculty-student relations more broadly, are crucial for student satisfaction and degree completion [3, 5, 7–9], yet little work has focused specifically on role model gender. (Some exceptions focus on the impact of role model gender on undergraduate students–not graduate students [10, 11]). Academic role models shape students’ confidence in their ability to succeed, and hence their persistence in academia [12, 13]. Without sufficient mentorship, students can become isolated from their academic communities, contributing to attrition [14]. Yet female role models are often few and far between for graduate students–limiting opportunities for mentorship to boost self-efficacy [15]. Women become increasingly underrepresented as they progress through academic ranks in many academic disciplines [16–19]. A 2017 NSF report found that one in three tenure-track or tenured science and engineering professors were women [4], and there has been little change in ‘the most severe gender divides in STEM areas’ for two decades [13].

We study exposure to two types of female role models: 1) authors whose work is assigned in Ph.D.-level syllabi and 2) faculty mentors during students’ academic careers. Though personally unknown to most students, authors appearing in syllabi act as role models; a citation in a syllabus signals that the author has conducted research worthy of emulation. However, scholars cite the work of female scholars infrequently, relative to women’s professional presence [20–24]. In political science, for example, women constituted only 19% of first authors of the citations in Ph.D.-level syllabi and reading lists; by contrast, women had authored 27% of articles in top ten political science journals and comprised 27% of US-based tenure-track political science faculty positions [23]. (In contract to some other disciplines, first-authorship in political science denotes greater prominence on a publication when author order deviates from alphabetical.) Similarly, undergraduate students across a wide range of disciplines see few women in their syllabi [25]. Faculty mentors serve as a second and more direct set of role models for students, as they shape students’ academic and career choices. Yet, as aforementioned, women are underrepresented as research faculty in many disciplines.

Although students have extensive exposure to cited authors and faculty mentors, students also find few female role models in numerous other academic venues [26–28]. Departments and conferences across academic fields may feature few female invited speakers, even relative to women’s representation in their respective fields [29–31]. Similarly, academic conferences not infrequently feature ‘manels’ (i.e. all-male panels) [32]. Scholarship also documents widespread gender citation gaps, in which articles cite male authors more frequently than female authors, even when accounting for women’s representation in a given field [13, 33–35].

Our study constitutes one of the first to investigate how these gaps affect students’ attitudes and academic success. Previous research has examined the impact of faculty and institutional biases on underrepresented students [12, 36–38], but not yet tested how faculty diversity affects students’ perceptions of their own academic abilities. Additionally, much prior work on graduate student success has focused either on admissions or attrition, with limited attention to the attitudes and experiences of current students [1, 3, 39]. Our novel, nationally-representative survey experiment therefore fills a critical gap in the literature on gender gaps in PhD training and student outcomes.

We focus on the dependent variable of self-efficacy, which previous scholars have defined as ‘beliefs in [one’s] ability to perform well in a variety of situations’ [40, 41]. Existing research suggests that self-efficacy predicts student retention, progress on dissertations, and doctoral completion [2, 42]. A principal reason self-efficacy predicts program outcomes is that students are among those most familiar with their own capabilities, challenges and barriers. As self-efficacy is domain-specific, our dependent variable comprises two sub-dimensions: students’ attitudes toward their future success in coursework and in their Ph.D. programs more generally.

Although some prior work indicates that gender is uncorrelated with academic self-efficacy [40], we expect that female academic role models should particularly influence the self-efficacy of female students. Female students are more likely than their male counterparts to view academic environments as overly competitive and aggressive, unsupportive in publishing and job-seeking, and lacking in female role models [27, 43, 44]. Women are also more likely than men to experience sexual harassment and violence in graduate school [38, 45]. Perhaps in light of these gender disparities, female graduate students and junior faculty report higher program and career satisfaction and have higher retention rates when engaged in gender-homophilous mentorship relationships (i.e. where mentor and mentee share the same gender) [10, 11, 46, 47]. These effects support the predictions of social identity theory that increasing identification with social groups’ affects individuals’ sense of self-worth [48–50]. We therefore expect that as female students seek out female role models, those role models will affect women’s subjective likelihood of academic success more strongly than that of men.

We also expect that attitudes toward diversity will shape students’ responses to female role models. Graduate students have increasingly observed and participated in scholarly and policy debates related to diversity–be they issues of representation, discrimination, bias, or inclusion [12, 13, 51–54]. These debates play out in scholarship, in professional venues such as conferences, and in struggles over university policies (e.g. maternity leave and spousal hiring) [55]. The media has also put a spotlight on reports of sexual harassment, particularly of female graduate students [45]. This public attention has likely prompted students to develop attitudes toward diversity that shape how they think about their own place and prospects in the academy.

Therefore, we expect that students who support diversifying academia–both syllabi and the professoriate–will tend to seek out female role models and to feel more efficacious upon exposure to gender-diverse environments. By contrast, we expect that students who are skeptical about diversifying the academy will feel less secure about their own trajectories in such environments. Role congruity theory of gendered leader evaluations [56] and social dominance theories [57] both suggest that individuals who do not value diversity will exhibit personal preference for male role models, while exposure to female role models may generate feelings of anxiety or insecurity. Because men exhibit higher levels of social dominance orientations than women do [58], and because dominant groups often view subordinate groups’ advancement in zero-sum terms [59], we expect men to react negatively to gender diversity more often than women.

To test the impact of female role models on self-efficacy, we employed focus groups and a survey of 297 students from the 50 top-ranked Ph.D. programs in one discipline: political science. We expect findings to be relevant for other academic disciplines due to the widespread underrepresentation of women across many disciplines, especially at the top faculty ranks. As of 2017, 32% of faculty in top 20 US political science departments were women [60], as were 35% of science and engineering faculty in the US [4]. The percentage female varied across disciplines–for instance, from 17% in engineering to 56% in psychology–yet men substantially outnumbered women in almost all STEM (Science, Technology, Engineering, and Mathematics) areas [4]. Women’s low representation among faculty limits female graduates students’ opportunities for same-gender mentoring, as female graduate students tend to outnumber female faculty. Women comprised approximately 47% of graduate students entering political science Ph.D. programs and 44% of students entering non-political science STEM disciplines in 2019 [4]. In addition, political science Ph.D. programs are structured in similar ways as other Ph.D. programs (e.g. coursework, comprehensive exams, candidacy, defense), although political science Ph.D. students are infrequently in a lab setting and funding does not typically depend on one’s Ph.D. advisor, or P.I.

We investigated Ph.D. students’ experiences with and reactions to two types of representation: a) female-authored scholarship in syllabi and b) female faculty role models. We also analyzed how students’ demographics and attitudes toward diversity affected their responses to the availability of female role models. The experimental component of our survey sought to test the impact of exposure to female authors in Ph.D.-level syllabi. We hypothesized that exposure to a syllabus with a higher percentage of female authors would increase female students’ self-efficacy (**hypothesis A**), and that attitudes toward diversity would condition students’ responses to syllabi with different proportions of female authors (**hypothesis B**). Non-experimental survey questions then investigated the *rate* and *consequences* of exposure to female role models in students’ own career networks. We hypothesized that female students would exhibit gender homophily, identifying a higher share of female role models than would be expected based on chance (**hypothesis C**). Further, we expected that having a higher number of role models would be associated with higher student self-efficacy (**hypothesis D)**; and that female role models would be more strongly associated with female students’ self-efficacy than would male role models (**hypothesis E**). Having more role models indicates that a student is successfully establishing more positive ties to professional academics, and it makes sense that students would feel that they would have a higher likelihood of success in their own academic trajectory as a result.

To summarize, we empirically investigated the relationship between exposure to female scholars and Ph.D. students’ self-efficacy. Our results suggest that these variables are indeed related, though not entirely as predicted. Analysis of an experiment revealed a particularly surprising result: exposure to more female-authored citations led certain groups of students to feel *less* likely to achieve academic success: namely, men, and students holding unfavorable views of diversity in academia. Nonetheless, correlational analysis of non-interventional survey results indicates that women and those with positive attitudes toward diversity seek out female role models. Importantly, having *more* role models–irrespective of the role model’s gender or race–correlates with positive academic self-efficacy.

## Materials and methods

The analysis below presents two sets of analyses, both drawn from a single survey. The survey was developed utilizing two focus groups conducted with political science and sociology Ph.D. students at a major research university not included in the survey sample. We recruited a nationally representative sample of political science Ph.D. students from the top 50 ranked political science Ph.D. programs in the United States to participate in an online survey, which was fielded between December 13 and December 16, 2019. A sample size of 300 (aiming for a minimum of 130 female respondents) was targeted, in order to achieve a statistical power of .8. Power calculations assumed a small effect of one-third of a standard deviation in the outcome; such an effect would be in line with prior interventions boosting student self-efficacy [61]. In total, we emailed invitations to approximately 2,000 students; we cut off survey administration at 300 students despite additional survey interest. In total, 308 students began the survey, and 297 completed it.

Both the qualitative and quantitative components of the data collection were registered as exempt with the Institutional Review Board (IRB) of the University of California, Irvine; and they were approved as exempt by the IRB of Iowa State University (IRB ID:19–502). Before beginning the focus group and survey, participants received an informed consent statement. In the focus group, they had an opportunity to discuss the consent statement, and then provided verbal consent; in the online survey, they provided informed consent by clicking to continue after reading the consent statement.

Table 1 presents the detailed demographic profile of our survey sample, as well as comparisons to the broader graduate student population in political science, from the American Political Science Association’s (APSA) 2019 statistics on the demographics of its general members. APSA’s data varies a bit from our own since many of its members are people who have already completed their PhDs. This difference is most notable in age distribution: APSA’s membership demographic skews much older than graduate students. The difference in age composition likely also helps to explain the differences in gender composition, since the percentage female within political science has been rising. On race and class markers, however, our survey is representative of the larger demographic composition of the field of political science.

**Table 1:** Characteristics of the sample.

	Our Survey	APSA 2019
Gender		
% Female	45.4%	37.4%
% Male	50.6%	62.4%
% Non-binary or Other	1.0%	0.2%
% NA	2.0%	No data
Age		
21–25	26.6%	1.6%
26–29	40.4%	5.9%
30–35	26.6%	18.6%
36–45	6.4%	29.9%
First in family to graduate from college?	19.5%	18%
Race/Ethnicity (multiple responses allowed)		
Black or African American	3.4%	4.9%
Hispanic, Latino, or Spanish	10.4%	5.9%
White	73.7%	75.3%
Asian	15.5%	9.5%
Middle Eastern or North African	3.0%	1.7%
Other	4.0%	2.3%
Has a dependent (i.e. elderly family member, child/children)	10.1%	No Data

Column 1 presents the percentage of our sample matching each demographic category. Column 2 presents the percentage of members of the American Political Science Association (APSA) in each category, using 2019 data. Note that APSA’s data includes people who have already completed their PhDs.

### Study 1

Our experiment proceeded as follows. After answering demographic questions, student participants read a randomly assigned version of an invented research methods syllabus titled “New Research Methods in Social Sciences.” The four versions of the syllabus were identical except for the assigned readings; the key randomization involved changing the first names of the authors of 20% of the readings from identifiably male to identifiably female. In our control conditions, only 10% of readings were female-authored; prior research documents that this is the mean representation of women on research methods syllabi in Ph.D. programs in political science [23, 24]. The treatment versions substantially raised the representation of female authors, to 30%. Research suggests that group behavior shifts when women reach a ‘critical mass’ of 15 to 30% in male-dominated environments [62, 63]. Note that in political science, most citation formats include fully-spelled out author first names, and so it was not unusual for student subjects to see authors’ first names instead of an initial. We selected all readings from our GRADS dataset of syllabi in graduate coursework in political science, as well as new literature in research methods. (For further information, visit: https://gradtraining.socsci.uci.edu/). To further prime students to notice scholars’ gender, writing prompts also referenced authors using gendered pronouns (e.g., “he” or “she”). In the S1 File, we present the full syllabus with all treatment conditions.

The authors and readings subject to experimental variation were artificial, in order to avoid changing scholars’ first names and genders in real citations. In addition, given uncertainty over how inserting invented readings into an otherwise realistic syllabus would affect student responses, we tested conditions that were fully and partially artificial. As a result, the four treatment conditions orthogonally varied both the percentage of the readings that were artificial (20% or 100%) and the percentage of the readings that were authored by women (10% or 30%):

- Condition 1 (10% women, 20% artificial): All readings authored by women were real, as were most of the readings authored by men. The 20% artificial citations received male first names.
- Condition 2 (30% women, 20% artificial): A third of the readings authored by women were real, as were all of the readings authored by men. The 20% artificial citations received female first names.
- Condition 3 (10% women, 100% artificial): All readings were artificial; the condition included the artificial citations from Conditions 1 and 2, giving those citations male first names.
- Condition 4 (30% women, 100% artificial): All readings were artificial; the condition included the artificial citations from Conditions 1 and 2, giving those citations female first names.

As we present in S5 Table in S1 File, our analysis revealed no impact of the full-versus-partially-artificial treatments. As a result, in the main text, we pool the two conditions with 10% women, as well as the two conditions with 30% women and present corresponding findings. As S1 Table in S1 File shows, the conditions are balanced on demographic variables, as well as relevant attitudes; we cannot dismiss the null hypothesis of balance at any standard level of statistical significance on any of the variables we tested.

**Fig 1:**
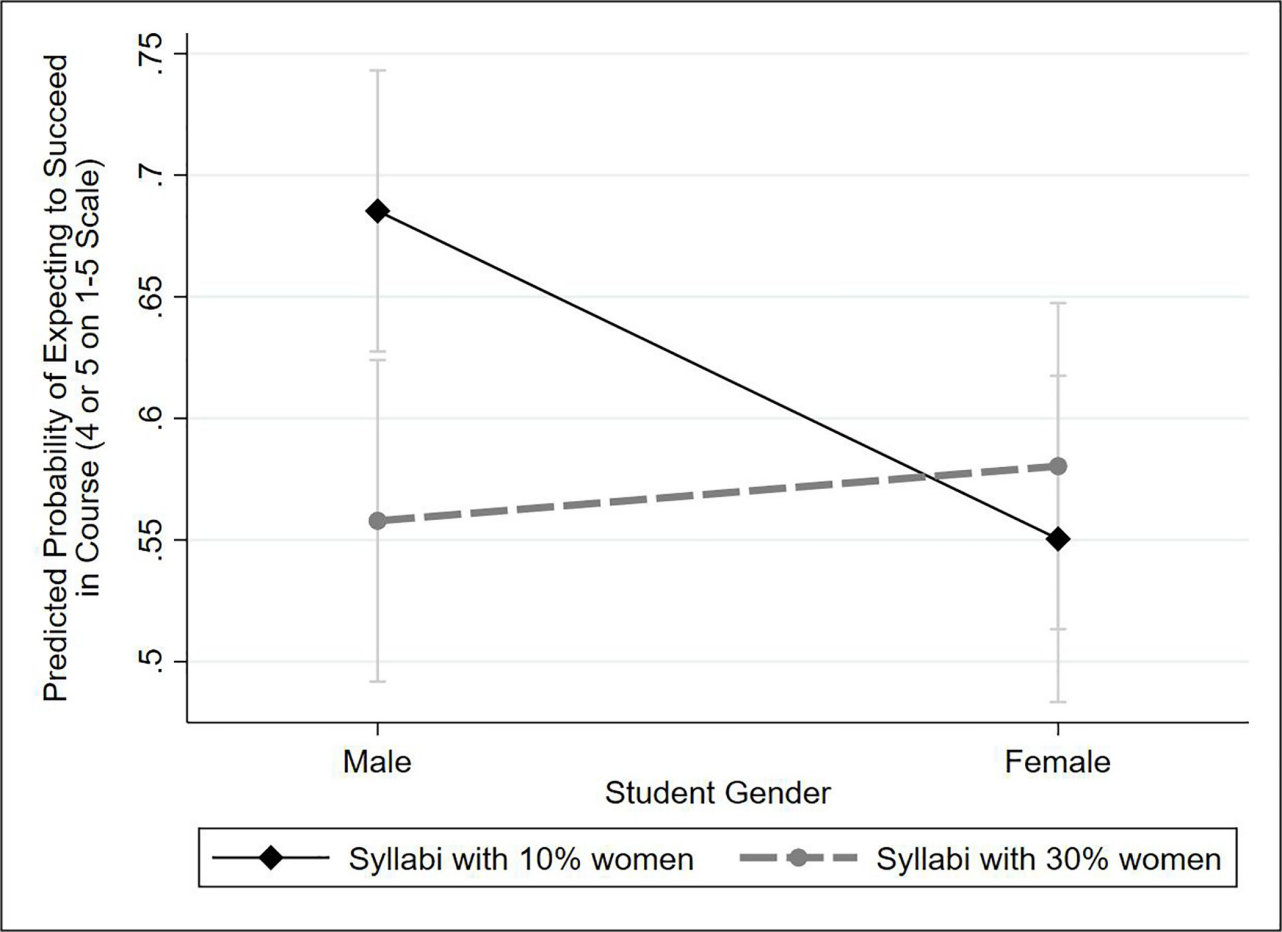
Impact of a gender-diverse syllabus on self-efficacy, by students’ gender. Results are from a logistic regression model predicting perceived likelihood of success in the course (coded as reporting a 4 or a 5 on a 1–5 scale), controlling for gender as well as orientations toward quantitative and qualitative methods, which are correlated with both gender and course-related self-efficacy (see S1 File for further discussion). The figure shows 76% confidence intervals; visual comparison of two 76% CIs is equivalent to a single 90% test [64, 65].

**Fig 2:**
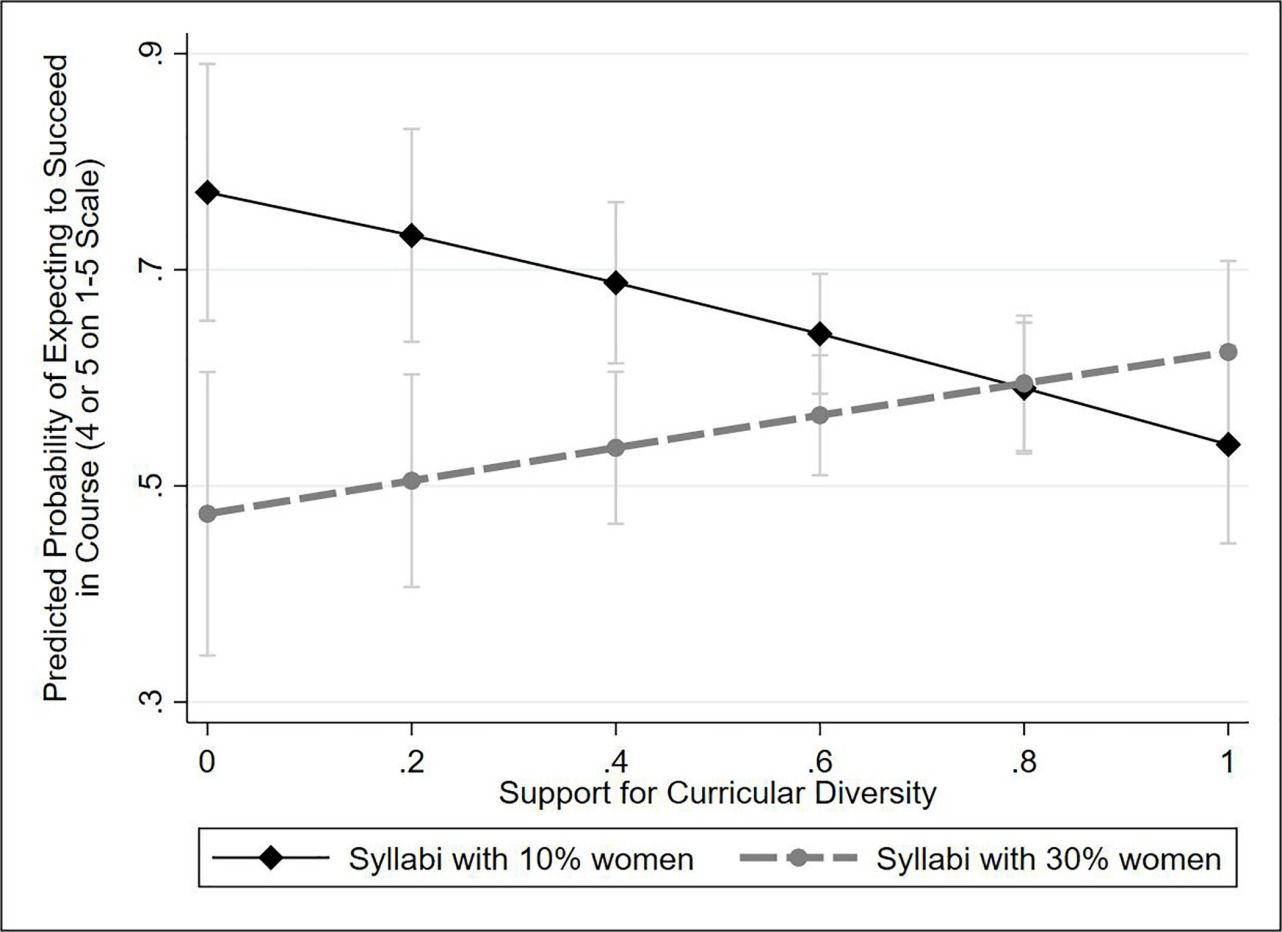
Impact of attitudes toward diversity on students’ responses to a gender-diverse syllabus. Results are from a logistic regression model predicting perceived likelihood of success in the course (coded as reporting a 4 or a 5 on a 1–5 scale), controlling for gender as well as orientations toward quantitative and qualitative methods, which are correlated with both gender and course-related self-efficacy (see S1 File for further discussion). The figure shows 84% confidence intervals; visual comparison of two 84% CIs is equivalent to a single 95% test [64, 65].

In analyzing results, our treatment indicator is a binary variable for the conditions with 10% versus 30% female-authored citations. The dependent variable, *course-related self-efficacy*, is based on a single question asking, “Do you feel as if you would be successful if you took this course?” Responses correspond to one of our two aforementioned sub-dimensions of self-efficacy: attitudes toward success in coursework. We recorded responses on a 1–5 scale, using the response “a great deal,” “a lot,” “a moderate amount,” “a little,” and “not at all.” In the primary analysis shown in Figs 1 and 2, the variable is dichotomized so that responses of “a great deal” and “a lot” are coded as “1,” and the remaining three responses are coded as “0”; the analysis uses logistic regression. In the S3 Table in S1 File presents the full multivariate models, using ordinal logistic regression.

In addition, the analysis includes several other variables. Respondents self-identified *student gender*; options were male, female, gender non-conforming or nonbinary, other and prefer not to answer. Only three students identified their gender as nonbinary or “other,” which is too small a sample for quantitative analysis. As a result, these students are excluded from the analysis. *Support for Curricular Diversity* is an index based on the mean of responses to two attitudinal statements, both on 1–7 scales running from strongly disagree to strongly agree: “I notice when a course syllabus has a lot of female-authored readings or very few female-authored readings” and “Professors should pay attention to gender balance when they write syllabi.” The Cronbach’s alpha reliability coefficient of the two-item index is .80. Scores are rescaled to run from 0 to 1.

Finally, because the syllabus relates to research methods, and gender correlates with attitudes toward research methods, much of the analysis controls for quantitative and qualitative orientation. *Quantitative orientation* is the mean of two items reading, “Please rate the following: Your interest in quantitative methods” and “Your ability in quantitative methods” (Cronbach’s alpha .79). Responses to each were on a scale running from 1 (“very low”) to 5 (“very high”); the mean of the two items was recoded to run from 0 to 1. *Qualitative orientation* is the mean of two equivalent items referring to qualitative methods, and likewise runs from 0 to 1 (Cronbach’s alpha .75).

### Study 2

In a non-interventional component of the survey, exposure to role models was measured with the following item: “We want to ask a few questions about academic role models. An academic role model is a person whom you admire professionally and whose research you admire. Graduate students often identify role models by reading that person’s research, seeing them teach or give a talk, or working for them as a research assistant. During your undergraduate or graduate training, have you identified any academic role models?” Response options allowed respondents to say that they had identified none, one, two, three to five, or six or more role models. The survey then asked respondents to think about their “top one or two academic role models.” The question followed: “Does this person (or does at least one of these people) identify as the same gender that you do?” We assume that a student has a female role model if a woman responds “yes” to this question, or a man responds “no.” We use logistic regression to predict whether a student has a female role model.

Our key outcome variable in the observational analysis is *program-related self-efficacy*, which we measured using an index (alpha = .83) based on the mean of responses to a battery of eight items. Student reported the extent to which they agreed with the following statements, on a 1–5 scale:

- I fit well in my PhD program.
- I am likely to finish my PhD.
- My research is likely to get published.
- My research is likely to be cited.
- Most of my fellow graduate students (who know me) think I am or will be successful.
- My fellow graduate students support me (non-financially).
- My PhD department supports me (non-financially).
- My advisor supports me (non-financially).

We analyze this variable in Table 3 using Ordinary Least Squares regression, with the dependent variable measured on a 1–5 scale.

Table 2 presents summary statistics for the key variables we have described; given that we often break our analysis out by student gender, we present means and standard deviations separately for each gender.

**Table 2:** Summary statistics for dependent variables and key independent variables, by respondent gender.

	Min	Max	Mean (Men)	Mean (Women)	Std Dev (Men)	Std Dev (Women)
Course-Related Efficacy	1	5	3.81	3.76	0.96	0.90
Program-Related Efficacy	0	1	0.72	0.71	0.17	0.16
Diversity Attitudes	0	1	0.56	0.75	0.29	0.26
Quantitative Orientation	0	1	0.69	0.63	0.24	0.25
Qualitative Orientation	0	1	0.49	0.59	0.24	0.22

## Results

To summarize our results, our experiment surprisingly revealed that the treatment (i.e. a gender-diverse syllabus) had no effect on female students’ self-efficacy, but instead significantly reduced male students’ self-efficacy. However, student attitudes toward diversity more strongly conditioned the treatment effect than did students’ own gender; exposure to a gender-diverse syllabus diminished the self-efficacy of students who did not value diverse role models. In the non-experimental component, we found positive links between female role models and female students’ self-efficacy: female students were disproportionately likely to have female role models, and academic role models correlated strongly (positively) with both women’s and men’s self-efficacy. Taken together, our results indicate that exposure to female role models correlates with students’ self-efficacy, but in ways shaped by students’ own experiences and views. As departments hire more women and diversify syllabi, some students are likely to feel less secure about their academic prospects. We discuss these results in more detail below.

### Study 1

Our experimental results did not support **hypothesis A**—that increasing women’s representation in syllabi would boost female students’ course-related self-efficacy. Rather, it made male students feel slightly *less* efficacious, as demonstrated in Fig 1. S3 Table in S1 File shows that student gender significantly conditioned the treatment effect, using an ordinal logistic regression analysis. Based on the logistic regression results reported in Fig 1, exposure to a 30% female syllabus lowers the predicted probability of male students rating their future success in the course as a ‘4’ or a ‘5’ from .69 to .56. By contrast, it raises female students’ probability of doing so to a statistically insignificant extent, from .55 to .58.

Consistent with **hypothesis B**, attitudes toward diversity significantly conditioned the treatment’s impact on self-efficacy (see S3 Table in S1 File, which uses ordinal logistic regression; the coefficient for the interaction is statistically significant at *p* < .05). Fig 2 below visualizes the relationship using logistic regression analysis and a binary dependent variable. Among students who were least supportive of diversity, exposure to a gender diverse syllabus lowered the predicted probability of reporting high course-related self-efficacy by about 30 percentage points, based on a logistic regression analysis. To put these results in context, the impact of the switch from a 10% to a 30% female syllabus among students least supportive of diversity is approximately equivalent to dropping from one standard deviation above the mean to one standard deviation below the mean on self-assessed quantitative ability (self-assessed quantitative ability is the strongest determinant of course-related self-efficacy). At the scale midpoint of support for diversity, the treatment had a small but statistically significant negative impact on course-related self-efficacy (the scale midpoint, however, is below the sample mean of .65 and median of .75). By contrast, the treatment increased the probability of reporting high course-related self-efficacy by a statistically insignificant 9 percentage points among students who most strongly supported diversity. Using ordinal logistic regression, exposure to a syllabus with 30% female authors lowers the predicted probability of rating one’s future success in the course as a ‘4’ or ‘5’ from .75 to .51 among students who are least supportive of diversity. By contrast, the exposure raises that probability from .55 to .62 among those who are most supportive of diversity. In S4 Table in S1 File, both interactions (treatment-by-gender and treatment-by-attitudes) are entered into a single ordinal logistic regression model. In those models, the treatment effect is largest among men with low support for diversity.

### Study 2

The non-experimental component of the survey sought to identify the distribution of female role models in students’ own professional networks, as well as the correlation between having female role models and self-efficacy. As detailed above, students reported whether they had none, one, two, three to five, or six or more academic role models, and then answered questions about the demographics of their “top one or two academic role models.” Consistent with **hypothesis C**, female students exhibited gender homophily in choosing role models. Prior research indicates that between 27 and 32 percent of faculty in Ph.D. granting departments are female (hence, given low numbers of gender nonbinary faculty, approximately 68 to 73 percent are male) [23, 29]. Thus, one can assess gender homophily by the extent to which female and male students differ from these percentages in reporting role models. Among students with role models, 77% of male students [95% CI: 70%, 85%] said they had a same-gender role model, and 66% [95% CI: 58%, 65%] of female students did likewise. Though the rate of homophily among male students does not differ significantly from what one would expect if role models were assigned randomly, the rate of gender homophily among women does. As a result, female faculty may experience higher demand from female students for mentorship–inadvertently exacerbating a gender gap in which female faculty face greater internal service demands than male faculty [66–68].

**Fig 3:**
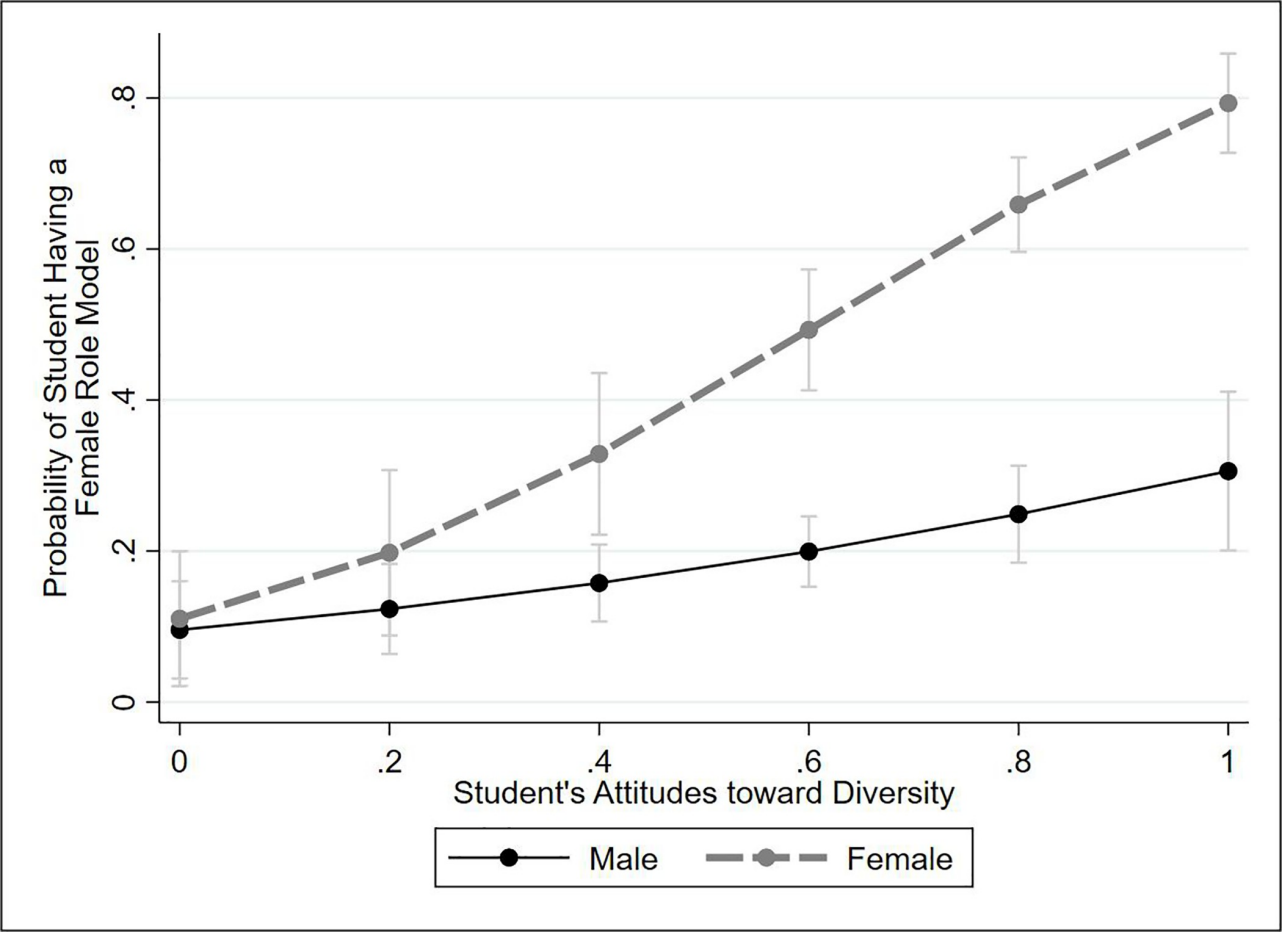
Impact of gender and attitudes toward diversity on having female role models. Results are predicted probabilities from a logistic regression model predicting likelihood of having a female role model (see S1 File for models). The figure shows 84% confidence intervals; visual comparison of two 84% CIs is equivalent to a single 95% test [64, 65].

Fig 3 visualizes the relationships among gender, attitudes toward diversity, and having female role models. Though our hypotheses had not specified an interaction between these variables, student gender and attitudes toward diversity interact powerfully to determine whether a student identifies a female role model. At the lowest level of support for diversity, the predicted probability that a male student identifies a female role model is 9.8%, and the probability of a female student doing so is 10.9%. Both rates are substantially below what one would expect if female role models were randomly assigned. At the highest levels of support for diversity, the genders diverge dramatically: male students have a 30.6% predicted probability of reporting a female role model, and female students a 79.6% probability. Female students exhibit statistically significant gender homophily when their support for diversity is at or above the scale midpoint in support for diversity (see S7 Table in S1 File).

We hypothesized that role models would be associated with self-efficacy (**hypothesis D**), and that female role models would be associated with particularly high self-efficacy for female students (**hypothesis E**). Table 3 presents an OLS model predicting an index of general program-related self-efficacy, by student gender, number of role models, role model gender, and the interaction of student and role model gender. (See S8 Table in S1 File for analysis controlling for a full range of demographic covariates, as well as controlling for quantitative and qualitative orientations and attitudes toward diversity.) The results support hypothesis D: faculty role models are associated with a substantial increase in students’ self-efficacy. Though 10% of students reported no role models and 7% reported six or more, the median student had between three and five role models. Moving from no role models to the sample median was associated with a gain in efficacy of 0.16 on the 0-to-1 scale, or one standard deviation of the dependent variable.

**Table 3:** Correlates of general academic self-efficacy (OLS).

	Coefficient	Standard Error	p-value <
Number of Role Models			
One Role Model	0.061	0.039	0.116
Two Role Models	0.122	0.036	0.001
Three to Five Role Models	0.161	0.034	0.001
Six or More Role Models	0.248	0.046	0.001
Female Role Model	-0.049	0.033	0.133
Female Student	-0.025	0.026	0.338
Female Student x Female Role Model	0.030	0.043	0.490
Constant	0.609	0.031	0.001
Number of Observations	286		
Adjusted R-Squared	0.111		

**Note:** Male student, male role model, and zero role models are the baseline categories. Models with various additional control variables are presented in *SI*: *Additional Analyses*.

However, the results do not support hypothesis E: role model gender did *not* matter for female students’ self-efficacy. Though many women preferred female role models, women who chose male role models had equally as high self-efficacy as women who chose female ones. While these results are contrary to our hypothesis, they are strikingly parallel to the findings from the experimental analysis, where increasing exposure to female role models did not significantly boost the self-efficacy either of women or students who already have attitudes of high support for diversity. Encouragingly, we also find no significant gender gaps in program-related self-efficacy in various analyses.

The findings for male students are also particularly important to note and discuss. Table 3 shows a negative baseline (non-interactive) coefficient for ‘female role model’ that is not statistically significant at standard levels. This means that male students with female role models reported slightly, but not statistically significantly, lower efficacy than did men with male role models. The reason this finding is noteworthy is that in follow-up analysis (see S7 Table in S1 File) controlling for a wide range of demographics as well as potentially relevant attitudes, the interaction term becomes statistically significant and negative (*b* = -.063, *p* = .047). Given that the coefficient varies in statistical significance across model specifications, we choose to report the simplest theoretically justifiable model here. Nonetheless, our findings do suggest the possibility of a slight gap in self-efficacy between men with male and female role models. Such a gap would again be in line with the negative impact of the treatment in the experimental analysis.

## Discussion

These results provide insights into the consequences of women’s underrepresentation for Ph.D. students’ success. We find that academic role models of all kinds bolster students’ self-efficacy, yet different students benefit from access to different role models. Some students eagerly seek out female role models and report having higher expectations of academic success when assigned to read female-authored scholarship. At the same time, other students take little active notice of gender diversity but feel less efficacious when assigned to read such scholarship. How students view diversity in academia shapes these responses.

First, students’ attitudes toward diversity conditioned their self-efficacy responses when exposed to female scholars in course syllabi. In addition, female students and students who support diversity sought out female role models in their environments. These findings provide clarity as to who benefits when Ph.D. programs adopt diversity initiatives: women and students who already believe that diversity is important. By implication, our evidence indicates initiatives to support diversity–for instance, hiring more female faculty and diversifying syllabi–do have concrete benefits for some graduate students.

Second, the results also reveal an unexpected phenomenon: male backlash. That is, some male students, notably ones with low support for curricular diversity, had lower expectations about academic success when confronted with increased gender diversity in syllabi. We had expected that female students would be most affected by seeing female role models, and that the effect would be positive. Instead, the dominant conclusion from our experimental analysis is that diversifying syllabi led some students to feel *less* likely to achieve academic success. Non-experimental analyses also offered results pointing in a similar direction, albeit less robustly: as discussed above, in some analyses, having a female role model is significantly correlated with lower self-efficacy among male students.

Why would some students react negatively to seeing 30% versus 10% female authors (meaning 70% versus 90% male authors)–on syllabi in their coursework? Additional studies are needed to confirm the finding in other fields and student populations, and to study the mechanisms underlying the effect, but we see several potential explanations. First, declining self-efficacy may result from a ‘backlash effect’ to female scholars’ increasing representation [69–71]. Scholars of gender define backlash as wielding coercive power to reinstate former hierarchies [72]. Sociologists, political scientists and scholars of management observe changes in men’s behavior in male-dominated organizations in response to women achieving a ‘critical mass’ of around 30%. Although a critical mass of women can yield substantive policy changes [73], it can also prompt resistance, opposition, and backlash among men, absent corresponding shifts in power structures [62, 71, 74–76].

Backlash could entail several related psychological mechanisms. Members of dominant social identity groups tend to view outgroups’ gains in ‘zero-sum’ terms, implying ingroup losses [59]; threats to identity and status can trigger fear and feelings of loss of control, as well as behaviors to reassert perceived dominance [77, 78]. Such behaviors are likely more common among individuals high in social dominance orientation (SDO) [57]. Hence, male students may have equated rising women’s representation in syllabi with their own group losing status and control, and responded with backlash. SDO could have triggered backlash among both women and men, but men on average have higher SDO [58].Second, some students might have interpreted the higher presence of women as a signal that the course was less academically rigorous. Implicit biases persist about women being less competent than men (and female-authored scholarship of lower quality) [12, 13, 79, 80]. Consequently, both male and female students holding such biases might have viewed training relying on gender diverse scholarship as being of lower value–leading them to question their own future success. A third possible explanation concerns gatekeeping and homophily in social networks [81]. Women’s growing presence in a field may lead male students to worry about losing access to benefits associated with all-male professional networks. If one views academia as an ‘old boys’ club’, such benefits would include access to male-dominant co-authorship, edited volumes, conference panels, workshops, and invited talks. Potential female mentors would lack access to such benefits. Exposure to a gender-diverse syllabus may have sent male students the signal that the field is more diverse than anticipated, meaning that benefits are far from guaranteed. The result would be a decrease in self-efficacy.

At the same time, our findings reveal that role models do raise students’ self-efficacy; the more role models, the better, irrespective of gender. Ph.D. students require mentorship to survive and thrive; mentorship is more important than any pre-existing factor (e.g. undergraduate GPA) in predicting retention [2]. If students cannot identify with at least one, and preferably more than one, academic role model, they may view the path to professional success as untenable. Although mentorship alone cannot change entrenched power disparities [82], it can buttress students’ belief in their own abilities, lifting their prospects for program completion.

We expect our findings to be generalizable to other disciplines. Among the physical, natural and social sciences, political science is just one of many disciplines with an interest in identifying factors undermining Ph.D. student retention. These disciplines similarly struggle with student retention challenges, diversity issues and debates, and the persistent underrepresentation of women as students and faculty. As many Ph.D. programs remain male-dominated, the psychological and social consequences of exposure to gender diversity should be epiphenomenal.

We improve upon literature on graduate student retention in several key ways. Our work identifies new and additional ways that diversity affects student experiences by concentrating on students’ own diversity-related attitudes and experiences. Additionally, analysis of our original data provides clear evidence that the number of academic role models matters greatly for graduate student experiences. Our research also offers other scholars survey instruments to improve predictions of students’ attrition risk.

One direction for future research is to investigate differential effects by race. Past work revealed that Ph.D. programs are racialized institutions [39], and that Ph.D. student satisfaction varies by race [36, 37, 83]. In prior studies, Black female graduate students as well as Latino and Latina graduate students reported receiving less effective mentorship than did other students, and being more likely to experience racism [84, 85]. Hence, future work should examine the ways that students’ own identities as well as their attitudes toward diversity influence responses to academic role models of color. Similarly, future work should focus on the experiences and reactions of transgender and nonbinary students. Yet another direction for future research is to examine how the type of course affects responses. For example, a backlash effect might not be observed in courses in which students anticipate seeing a high proportion of female authors.

Scientists have a clear interest in retaining the best potential researchers, regardless of their gender or racial/ethnic identity [12]. Scholarship has therefore investigated numerous factors that contribute to “leaks” in the pipeline of women and underrepresented minorities. Our findings have several practical implications for departments. First, hiring more female faculty, expanding female faculty mentorship, and diversifying syllabi to include more women will affect how students view their own career trajectories. Second, programs may need to seek to mitigate student backlash against diversity initiatives in order to support all students’ success. In this regard, applied research should investigate the etiology of students’ attitudes toward diversity. Presumably, students enter Ph.D. programs with predispositions toward diversity initiatives, yet we know little about whether or how Ph.D. programs can shift these attitudes [86, 87]. One promising course of action may be to supplement standard first year course readings with studies on gender and racial disparities and implicit biases in academia.

## Supporting information

S1 File Methods and analyses is a single file containing all supplemental information. (DOCX)
