# Supplementary material for: Having female role models correlates with PhD students’ attitudes toward their own academic success
Source: PLoS One. 2021 Aug 18;16(8):e0255095. doi: 10.1371/journal.pone.0255095 (PMC8372968; doi:10.1371/journal.pone.0255095)
Supplement: S1 File — (DOCX) [file pone.0255095.s001.docx]

**S1 File**

for “Having female role models correlates with PhD students’ attitudes toward their own academic success”

1. **Further Details on Methods**

**A. Focus Groups**

Twelve female and three male Ph.D. students in political science and sociology were interviewed in two single-gender focus groups at a leading research university in November 2019. The purpose was to develop and test questions to be utilized in the quantitative, nationwide survey.

*Protocol and Administration.* Questions focused on students’ role models, how they select courses, and their past experiences with professors. To preserve student confidentiality and minimize the risk that subjects and moderators would know one another, we hired two female and two male focus group moderators who were graduate students from non-political science disciplines (specifically, sociology and anthropology) who were studying at a different university in the same large metropolitan area.

*Recruitment and Inclusion and Exclusion Criteria.* Subjects were recruited via emails sent by departmental administrative staff; participants were incentivized to participate with $15 Amazon gift certificates, free pizza, and free sodas. All students identifying enrolled in the respective Ph.D. programs in the two departments were eligible for inclusion in the study, and no other students were eligible for inclusion.

*Demographics.* Moderators collected demographic information from the focus group participants, including age, race/ethnicity, family status, whether or not a student was a first-generation college graduate, and parental status. Past research has confirmed that graduate student retention varies along these demographic lines. Twelve ethnically diverse students attended the all-female group (58% non-white, 42% white), and three white male students attended the all-male group. The demographic characteristics of focus group participants varied somewhat from the population of the specific Ph.D. program; the program itself had 53% female students and 29% white students.

*Analysis and subject confidentiality.* Detailed summaries from the focus groups were read and analyzed for key themes related to graduate student satisfaction and retention. A draft of the quantitative survey instrument was then modified based on analysis of students’ language and themes. To de-identify the students, each participant received an identity number that was used in note taking and transcripts. One of the PIs was physically on-site at the university during the time that the focus groups were conducted in case moderators had any questions or any issues were to arise. However, to protect subject confidentiality, the on-site PI did not enter either of the two rooms and had no interaction with the subjects during the administration of the focus groups.

1. **Recruitment and Inclusion and Exclusion Criteria for Quantitative Study**

Participants were recruited from the top 50 PhD programs in the United States, according to the 2019 *US World News Report* list, which is widely accepted as an authority on academic program rankings. We developed our list of student subjects to recruit via publicly-available departmental websites, as well as via emails to administrative support staff. In total, we emailed invitations to approximately 2,000 students and these invitations offered a $15 gift certificate for survey completion. All students whom we were able to identify as enrolled in the respective programs, and for whom we were able to obtain email addresses, were eligible for inclusion in the study. All other potential respondents were excluded. Given budget limitations, we cut off survey administration at 300 students despite additional survey interest. In total, 308 students began the survey, and 297 completed it.

1. **Sample Balance on the Syllabus Treatment**

Before analyzing our survey experiment, we tested for balance across the two theoretically relevant treatment conditions by examining whether the randomly selected samples differed in a statistically significant manner on any attitudinal or demographic variable. As Table S1 shows, the conditions are balanced on a wide range of demographic variables, as well as relevant attitudes; we cannot dismiss the null hypothesis that the samples are balanced at any standard level of statistical significance on any of the variables we tested.

Table S1. Balance on Demographics and Relevant Attitudes, by Experimental Condition

|  | µ_1_ (10% Female Conditions) | µ_2_ (30% Female Conditions) | p (µ_1_ = µ_2_) |
| --- | --- | --- | --- |
| Quantitative Orientation | 0.64 | 0.66 | 0.43 |
| Qualitative Orientation | 0.55 | 0.54 | 0.66 |
| Diversity Attitudes | 0.65 | 0.65 | 0.96 |
| Female | 0.46 | 0.49 | 0.61 |
| Year in Program | 3.93 | 3.75 | 0.40 |
| Age Bracket | 2.19 | 2.07 | 0.25 |
| First Generation | 0.18 | 0.21 | 0.47 |
| White | 0.75 | 0.73 | 0.66 |
| Has Dependent | 0.09 | 0.11 | 0.63 |

Note: Cell entries in the first and second column represent the mean of each respective variable within the two treatment conditions. P-values in the third column represent a test of the null hypothesis that the means are equal.

1. **Additional Analyses**
2. **Necessity of Controls for Quantitative and Qualitative Orientations**

Much of the analysis controls for orientations toward quantitative and qualitative methods (see the main text for variable coding). As Table S2 shows, methods orientations are correlated with both gender and with academic self-efficacy of Ph.D. students in political science. Hence, these controls enable us to assess the effects of the treatment on academic self-confidence among women and men, net of gender differences in students’ confidence in their quantitative and qualitative abilities.

Table S2. Correlations among Gender, Methods Orientations, and Efficacy

|  | Female | Quantitative Orientation | Qualitative Orientation | Course-Related Efficacy | Program-Related Efficacy |
| --- | --- | --- | --- | --- | --- |
| Female | 1.000 |  |  |  |  |
| Quantitative Orientation | -0.115* | 1.000 |  |  |  |
| Qualitative Orientation | 0.201* | -0.265* | 1.000 |  |  |
| Course-Related Efficacy | -0.028 | 0.316* | -0.060 | 1.000 |  |
| Program-Related Efficacy | -0.036 | 0.333* | 0.015 | 0.219* | 1.000 |

Notes: Cell entries represent pairwise Pearson correlation coefficients. * indicates p < .05.

1. **OLS Regression Results Corresponding to Analysis of Experiment**

We present the full analysis from Figures 1 and 2 in Table S3. While the figures in the main text rely on logistic regression, the models below analyze the dependent variable on its original five-point scale, using ordinal logistic regression. Model 1 corresponds to Figure 1 in the main text; Model 2 corresponds to Figure 2 in the main text. Finally, a model including both interactions simultaneously (also discussed in the main text) is shown in Table S4.

Table S3. Full Interactive Results from Experiment (Ordinal Logistic Regression Results)

|  | Model 1  (corresponds to Figure 1) | | |  | Model 2  (corresponds to Figure 2) | | |  |
| --- | --- | --- | --- | --- | --- | --- | --- | --- |
|  | Coefficient | Standard Error | p-value < |  | Coefficient | Standard Error | p-value < | |
| Treatment (30% women) | -0.609 | 0.309 | 0.049 |  | -1.239 | 0.554 | 0.025 | |
| Female Student | -0.431 | 0.313 | 0.168 |  | -0.007 | 0.240 | 0.978 | |
| Treatment * Female Student | 0.816 | 0.453 | 0.072 |  |  |  |  | |
| Diversity Attitudes |  |  |  |  | -1.043 | 0.602 | 0.083 | |
| Treatment * Diversity Attitudes |  |  |  |  | 1.557 | 0.777 | 0.045 | |
| Quantitative Orientation | 3.554 | 0.538 | 0.001 |  | 3.545 | 0.540 | 0.001 | |
| Qualitative Orientation | 0.586 | 0.523 | 0.263 |  | 0.775 | 0.527 | 0.142 | |
| Cutpoint 1 | -1.749 | 0.621 |  |  | -2.135 | 0.697 |  | |
| Cutpoint 2 | -1.169 | 0.574 |  |  | -1.556 | 0.655 |  | |
| Cutpoint 3 | 1.892 | 0.550 |  |  | 1.502 | 0.629 |  | |
| Cutpoint 4 | 3.567 | 0.581 |  |  | 3.179 | 0.654 |  | |
| Adjusted R-Squared | 285 |  |  |  | 285 |  |  | |

Table S4. Full Results from Experimental Analysis, Including Interactions by Student Gender and Diversity Attitudes (Ordinal Logistic Regression Results)

|  | Coefficient | Standard Error | p-value < |
| --- | --- | --- | --- |
| Treatment (30% women) | -1.293 | 0.557 | 0.020 |
| Female Student | -0.279 | 0.332 | 0.400 |
| Treatment * Female Student | 0.571 | 0.483 | 0.237 |
| Diversity Attitudes | -0.853 | 0.621 | 0.170 |
| Treatment * Diversity Attitudes | 1.224 | 0.826 | 0.138 |
| Quantitative Orientation | 3.573 | 0.539 | 0.001 |
| Qualitative Orientation | 0.669 | 0.534 | 0.211 |
| Cutpoint 1 | -2.192 | 0.698 |  |
| Cutpoint 2 | -1.611 | 0.655 |  |
| Cutpoint 3 | 1.460 | 0.629 |  |
| Cutpoint 4 | 3.143 | 0.653 |  |
| Number of Observations | 285 |  |  |

1. **Analysis of Full-Versus-Partially-Artificial Syllabus Treatment**

As described in the main text, the survey experiment included a treatment orthogonal to the gender manipulation, in which we varied the percentage of citations that were artificial, from 20% to 100%. The purpose was to assess whether the necessary inclusion of artificial citations as a result of the gender manipulation affected students’ responses. In a wide range of analyses, we found no statistically significant effect from manipulating the percentage of the citations that were artificial. Among both men and women, across the range of attitudes toward diversity, and in the pooled sample as a whole, increasing the number of artificial citations had no impact on responses. The mean of the dependent variable (course-related self-efficacy) is 3.84 in the “all artificial” condition and 3.78 in the “partially artificial” condition (*p*=.45). Table S5 presents results from interactive models of this treatment by gender and by diversity attitudes, controlling for quantitative and qualitative orientations.

Table S5. Analysis of the Effect of Varying the Percentage of the Syllabus Including Artificial Citations, by Student Gender and Diversity Attitudes (OLS Results)

|  | Model 1 | | |  | Model 2 | | |
| --- | --- | --- | --- | --- | --- | --- | --- |
|  | Coefficient | Standard Error | p-value < |  | Coefficient | Standard Error | p-value < |
| Treatment (All Artificial Names) | -0.040 | 0.144 | 0.779 |  | 0.209 | 0.258 | 0.417 |
| Female Student | -0.110 | 0.148 | 0.458 |  | 0.024 | 0.112 | 0.831 |
| Treatment * Female Student | 0.245 | 0.209 | 0.243 |  |  |  |  |
| Diversity Attitudes |  |  |  |  | 0.051 | 0.261 | 0.846 |
| Treatment * Diversity Attitudes |  |  |  |  | -0.202 | 0.361 | 0.576 |
| Quantitative Orientation | 1.345 | 0.220 | 0.001 |  | 1.333 | 0.221 | 0.001 |
| Qualitative Orientation | 0.109 | 0.231 | 0.637 |  | 0.139 | 0.236 | 0.555 |
| Constant | 2.857 | 0.235 | 0.001 |  | 2.754 | 0.277 | 0.001 |
| Number of Observations | 286 |  |  |  | 286 |  |  |
| Adjusted R-Squared | 0.11 |  |  |  | 0.10 |  |  |

1. **Correlates of Reporting Female Role Models**

In Table S6, we assess correlates of reporting that one has a primary role model who is female (note that the analysis includes all respondents, including those reporting no role models of either gender). The first model presents the simplest, non-interactive analysis. The second model corresponds to Figure 3 in the main text. The third model controls for a wide variety of additional covariates that might possibly also affect choices of role models.

Table S6. Correlates of Reporting a Female Primary Role Model

|  | (1) | (2) | (3) |
| --- | --- | --- | --- |
| Female Student | 1.562* | 0.141 | 0.090 |
|  | *(0.283)* | *(0.837)* | *(0.876)* |
| Attitudes toward Diversity | 2.488* | 1.427^ | 1.432^ |
|  | *(0.563)* | *(0.774)* | *(0.807)* |
| Female x Attitudes toward Diversity |  | 2.039^ | 2.043^ |
|  |  | *(1.133)* | *(1.185)* |
| Quantitative Orientation |  |  | -0.214 |
|  |  |  | *(0.623)* |
| Qualitative Orientation |  |  | 0.550 |
|  |  |  | *(0.681)* |
| Year in Program |  |  | -0.039 |
|  |  |  | *(0.104)* |
| Age Bracket |  |  | 0.140 |
|  |  |  | *(0.226)* |
| First Generation Status |  |  | -0.502 |
|  |  |  | *(0.372)* |
| White |  |  | -0.290 |
|  |  |  | *(0.350)* |
| Has Dependents |  |  | -0.235 |
|  |  |  | *(0.549)* |
| Constant | -2.940* | -2.246* | -2.198* |
|  | *(0.435)* | *(0.530)* | *(0.848)* |
| Number of Observations | 286 | 286 | 286 |

Notes: Logistic regression coefficients shown; standard errors in parentheses. Coefficients are statistically significant at ^ p < .10; * p < .05.

1. **Correlates of Program-Related Self-Efficacy**

In Table S7, we present a full model corresponding to Table 1, controlling for demographics, quantitative and qualitative orientations, and support for diversity. As discussed in the main text, this analysis shows that men with a female primary role model have significantly lower levels of program-related self-efficacy than do men with a male primary role model. In addition, the non-interactive coefficient for *female student* indicates that women may have slightly lower levels of program-related self-efficacy when mentored by men (*p*=.099).

Table S7. Correlates of General Academic Self-Efficacy (OLS)

|  | Coefficient | Standard Error | P>t |
| --- | --- | --- | --- |
| Number of Role Models |  |  |  |
| One Role Model | 0.044 | 0.038 | 0.247 |
| Two Role Models | 0.100 | 0.036 | 0.005 |
| Three to Five Role Models | 0.135 | 0.033 | 0.001 |
| Six or More Role Models | 0.201 | 0.046 | 0.001 |
| Female Role Model | -0.063 | 0.032 | 0.047 |
| Female Student | -0.042 | 0.025 | 0.099 |
| Female Student x Female Role Model | 0.068 | 0.042 | 0.101 |
| Year in Program | 0.001 | 0.007 | 0.882 |
| Age 26-29 | -0.003 | 0.025 | 0.909 |
| Age 30-35 | -0.009 | 0.032 | 0.780 |
| Age 36-45 | -0.024 | 0.050 | 0.628 |
| First Generation Status | -0.033 | 0.022 | 0.138 |
| White | 0.015 | 0.022 | 0.491 |
| Has Dependents | 0.009 | 0.034 | 0.798 |
| Quantitative Orientation | 0.227 | 0.039 | 0.001 |
| Qualitative Orientation | 0.069 | 0.042 | 0.099 |
| Diversity Attitudes | 0.028 | 0.035 | 0.431 |
| Constant | 0.426 | 0.053 | 0.001 |
| *Number of Observations* | *286* |  |  |
| *Adjusted R-Squared* | *0.193* |  |  |

1. **Survey Experiment Instrument**

**Note:** The following is a copy of the syllabus that respondents viewed as part of the survey experiment. Respondents were randomly assigned one of four treatment conditions. Syllabi were identical except for the different treatment conditions that are indicated by different colored highlighting. For example, the syllabus with the treatment condition of “10% female-authored/mostly real names” displayed the specific citations listed under the orange highlighting. By contrast, the treatment condition of “30% female-authored/mostly real names” displayed those specific citations listed under the blue highlighting, etc. In addition, names highlighted in yellow in the writing prompts were varied across conditions.

**New Research Methods in the Social Sciences**

Fall 2019

**Course Objective**

This class will serve as a survey of the diverse array of interesting and cutting-edge research methods in the social sciences, ranging from topics such as machine learning and regression trees to ethnography. This class is not designed to acquaint you with heuristic concepts, rather, it is designed to familiarize you with a broad range of qualitative, quantitative, and multi-method research techniques which may serve you in your own work.

**Course Expectations**

Every week, one student will be responsible for leading discussion of a method, under my moderation. (We will select our weeks on the first class meeting.) Students who are not leading discussion that week are responsible for a 2 page memo on that week’s topic. Included with that memo should be at least three questions to contribute to the class discussion.

Each student will be responsible for a final research paper (20-25 pages), where they create a research design with an original research question relevant to their work, and utilizing one of the methods we have discussed in class. Papers are due to me via email on the Tuesday of finals week.

**Course Schedule**

**Week 1**

**Introduction to Course: Qualitative, Quantitative, or both?**

**Readings:**

*10% Women/Mostly Real Names Condition:*

- James Mahoney and Gary Goertz, “A Tale of Two Cultures: Contrasting Quantitative and Qualitative Research,” *Political Analysis* 14.3 (2006), pp. 227-249.
- Jason Seawright. Multi-Method Social Science: Combining Qualitative and Quantitative Tools. New York: Cambridge University Press, 2016. (Chapters 1-4)
- Aaron Castel. Multimethod research: A synthesis of styles. Thousand Oaks, CA, US: Sage Publications, 2015.

*30% Women/Mostly Real Names Condition:*

- James Mahoney and Gary Goertz, “A Tale of Two Cultures: Contrasting Quantitative and Qualitative Research,” *Political Analysis* 14.3 (2006), pp. 227-249.
- Jason Seawright. Multi-Method Social Science: Combining Qualitative and Quantitative Tools. New York: Cambridge University Press, 2016. (Chapters 1-4)
- Angela Castel. Multimethod research: A synthesis of styles. Thousand Oaks, CA, US: Sage Publications, 2015.

*10% Women/All Fake Names Condition:*

- Jared Maltzer and Gary Miller, “Comparing and Contrasting Quantitative and Qualitative Research,” *Political Analysis* 14.3 (2006), pp. 227-249.
- Charles Gregory. Combining Qualitative and Quantitative Tools for Multi-Method Social Science. New York: Cambridge University Press, 2016. (Chapters 1-4)
- Aaron Castel. A synthesis of styles: Multimethod Approaches to Research. Thousand Oaks, CA, US: Sage Publications, 2015.

*30% Women/All Fake Names Condition:*

- Jared Maltzer and Gary Miller, “Comparing and Contrasting Quantitative and Qualitative Research,” *Political Analysi*s 14.3 (2006), pp. 227-249.
- Charles Gregory. Combining Qualitative and Quantitative Tools for Multi-Method Social Science. New York: Cambridge University Press, 2016. (Chapters 1-4)
- Angela Castel. A synthesis of styles: Multimethod Approaches to Research. Thousand Oaks, CA, US: Sage Publications, 2015.

Reading Memo: In your paper, make sure to contrast [Aaron/Angela] Castel’s approach to research design with [Jason Seawright’s/Charles Gregory’s].

**Week 2**

**Machine Learning in Social Sciences**

**Readings**

*10% Women/Mostly Real Names Condition:*

- David Castaños, et al. "Comparing random forest with logistic regression for predicting class-imbalanced civil war onset data." *Political Analysis* 24.1 (2015): 87-103.
- Paul Limer, et al. "Topic models for open ended survey responses with applications to experiments." *American Journal of Political Science* (2014).
- James Burrell. “How the machine ‘thinks’: Understanding opacity in machine learning algorithms.” *Big Data & Society* (2016).

*30% Women/Mostly Real Names Condition:*

- David Castaños, et al. "Comparing random forest with logistic regression for predicting class-imbalanced civil war onset data." *Political Analysis* 24.1 (2015): 87-103.
- Paul Limer, et al. "Topic models for open ended survey responses with applications to experiments." *American Journal of Political Science* (2014).
- Jasmine Burrell. “How the machine ‘thinks’: Understanding opacity in machine learning algorithms.” *Big Data & Society* (2016).

*10% Women/All Fake Names Condition:*

- David Castaños, et al. "Comparing random forest with regression for predicting civil war onset." *Political Analysis* 24.1 (2015): 87-103.
- Paul Limer, et al. "Topic models for survey responses with experimental application." *American Journal of Political Science* (2014).
- James Burrell. “How the machine ‘understands’: opacity in machine learning algorithms.” *Big Data & Society* (2016).

*30% Women/All Fake Names Condition:*

- David Castaños, et al. "Comparing random forest with regression for predicting civil war onset." *Political Analysis* 24.1 (2015): 87-103.
- Paul Limer, et al. "Topic models for survey responses with experimental application." *American Journal of Political Science* (2014).
- Melinda Burrell. “How the machine ‘understands’: opacity in machine learning algorithms.” *Big Data & Society* (2016).

Reading Memo: How can machine learning be applied? Please be sure to address the differences between abstract and applied machine learning (as described in the David Castaños et al and [James/Jasmine] Burrell papers, respectively).

**Week 3**

**Sampling and Survey Design**

**Readings**

*10% Women/Mostly Real Names Condition:*

- David de Vaus, Surveys in Social Research. London: Routledge, 2013. (Chapters 1-4, 6).
- James M.T. Collins, “A Typology of Mixed Methods Sampling Designs in Social Science Research.” *The Qualitative Report* (2007).
- Christine Brinkman Bhutta, “Not by the book: Facebook as a Sampling Frame.” *Sociological Methods and Research* (2012).

*30% Women/Mostly Real Names Condition:*

- David de Vaus, Surveys in Social Research. London: Routledge, 2013. (Chapters 1-4, 6).
- James M.T. Collins, “A Typology of Mixed Methods Sampling Designs in Social Science Research.” *The Qualitative Report* (2007).
- Christine Brinkman Bhutta, “Not by the book: Facebook as a Sampling Frame.” *Sociological Methods and Research* (2012).

*10% Women/All Fake Names Condition:*

- David de Varney, Surveys and Social Research. London: Routledge, 2013. (Chapters 1-4, 6).
- James M.T. Collins, “Mixed Methods Sampling Designs in Social Science Research: A Typology” *The Qualitative Report* (2007).
- Christina M. Patel, “ Facebook as a Sampling Frame: Going by the Book.” *Sociological Methods and Research* (2012).

*30% Women/All Fake Names Condition:*

- David de Varney, Surveys and Social Research. London: Routledge, 2013. (Chapters 1-4, 6).
- James M.T. Collins, “Mixed Methods Sampling Designs in Social Science Research: A Typology” *The Qualitative Repor*t (2007).
- Christina M. Patel, “Facebook as a Sampling Frame: Going by the Book.” *Sociological Methods and Research* (2012).

Reading Memo: How does [Christine Bhutta/Christina Patel] utilize the concepts that [David de Vaus/David de Varney] lays out in his book?

**Week 4**

**ArcGIS**

**Readings**

*10% Women/Mostly Real Names Condition:*

- Lazaro M. Scott and Mark V. Jenkis, “Spatial Statistics in ArcGis.” *Handbook of Applied Spatial Analysis* (2010).
- Richard P. Green and James B. Pick, Exploring the Urban Community: A GIS Approach. Prentice Hall, 2012. (Chaps 1-3)
- Curt Weidmann and Julian Smith. “Social Media Location Intelligence: The Next Privacy Battle.” *International Journal of Geoinformatics* (2013).

*30% Women/Mostly Real Names Condition:*

- Lazaro M. Scott and Mark V. Jenkis, “Spatial Statistics in ArcGis.” *Handbook of Applied Spatial Analysis* (2010).
- Linda Loubert. Exploring the Urban Community: A GIS Approach. Prentice Hall, 2012. (Chaps 1-3)
- Curt Weidmann and Julian Smith. “Social Media Location Intelligence: The Next Privacy Battle.” *International Journal of Geoinformatics (2*013).

*10% Women/All Fake Names Condition:*

- Lazaro M. Scott and Mark V. Jenkis, “ArcGis and Spatial Statistics.” *Handbook of Applied Spatial Analysis* (2010).
- Richard P. Greensley and James B. Pickery, Exploring the Rural Community: A GIS Approach. Prentice Hall, 2012. (Chaps 1-3)
- Curt Saidesmann and Julian Markney. “Social Media Intelligence: The Next Location Privacy Battle.” *International Journal of Geoinformatics* (2013).

*30% Women/All Fake Names Condition:*

- Lazaro M. Scott and Mark V. Jenkis, “ArcGis and Spatial Statistics.” *Handbook of Applied Spatial Analysis* (2010).
- Leanna P. Greensley, Exploring the Rural Community: A GIS Approach. Prentice Hall, 2012. (Chaps 1-3)
- Curt Saidesmann and Julian Markney. “Social Media Intelligence: The Next Location Privacy Battle.” *International Journal of Geoinformatics* (2013).

Reading Memo: What are the differences in [Richard Green and James Pick’s/Linda Loubert’s] approaches to using ArcGIS versus [Curt Weidmann and Julian Smith’s/Curt Saidesmann and Julian Markney]?

**Week 5**

**Immersive Ethnography**

**Readings**

*10% Women/Mostly Real Names Condition:*

- Edward Schatz, Political Ethnography: What Immersion Contributes to the Study of Power. Chicago and London: University of Chicago Press, 2009. (Chap 1)
- Timothy Pachirat, Every Twelve Seconds: Industrialized Slaughter and the Politics of Sight. Yale University Press, 2013.
- Kevin J. Brown and Frederick D. Weil. “Strangers in the Neighborhood: Violence and Neighborhood Boundaries.” *Journal of Contemporary Ethnography* (2019).

*30% Women/Mostly Real Names Condition:*

- Jessica Schultz, Political Ethnography: What Immersion Contributes to the Study of Power. Chicago and London: University of Chicago Press, 2009. (Chap 1)
- Timothy Pachirat, Every Twelve Seconds: Industrialized Slaughter and the Politics of Sight. Yale University Press, 2013.
- Kevin J. Brown and Frederick D. Weil. “Strangers in the Neighborhood: Violence and Neighborhood Boundaries.” *Journal of Contemporary Ethnography* (2019).

*10% Women/All Fake Names Condition:*

- Edwin Schultz, Political Ethnography: What Immersion Changes in the Study of Violence. Chicago and London: University of Chicago Press, 2009. (Chap 1)
- Charles M. Pachno, Industrialized Labor and the Politics of Class. Yale University Press, 2013.
- Kevin J. Biggs and Frederick D. Weil. “There goes the Neighborhood: Violence and Neighborhood Boundaries.” *Journal of Contemporary Ethnography* (2019).

*30% Women/All Fake Names Condition:*

- Jessica Schultz, Political Ethnography: What Immersion Changes in the Study of Violence. Chicago and London: University of Chicago Press, 2009. (Chap 1)
- Charles M. Pachno, Industrialized Labor and the Politics of Class. Yale University Press, 2013.
- Kevin J. Biggs and Frederick D. Weil. “There goes the Neighborhood: Violence and Neighborhood Boundaries.” *Journal of Contemporary Ethnography* (2019).

Reading Memo: In your paper, you should look at the different ways in which [Edward Schatz/Jessica Schultz/Edwin Schultz], [Timothy Pachirat/Charles M. Pachno], [Kevin Brown and Frederick Weil/Kevin J. Biggs and Frederick D. Weil] approach the concept of immersive ethnography.

**Week 6**

**Qualitative Analysis Software: An Introduction**

**Readings**

*10% Women/Mostly Real Names Condition:*

- Darnell C. Watkins, “Rapid and Rigorous Qualitative Data Analysis: The “RADaR” Technique for Applied Research.” *International Journal for Qualitative Methods* (2017).
- Michael J. Belatto, “Data Analysis Methods for Qualitative Research.” Th*e Qualitative Report* (2018).
- Nora Kalpokaite, “Demystifying Qualitative Data Analysis for Novice Qualitative Researchers.” *The Qualitative Report* (2018).

*30% Women/Mostly Real Names Condition:*

- Darnell C. Watkins, “Rapid and Rigorous Qualitative Data Analysis: The “RADaR” Technique for Applied Research.” *International Journal for Qualitative Methods* (2017).
- Michael J. Belatto, “Data Analysis Methods for Qualitative Research.” *The Qualitative Report* (2018).
- Nora Kalpokaite, “Demystifying Qualitative Data Analysis for Novice Qualitative Researchers.” *The Qualitative Report* (2018).

*10% Women/All Fake Names Condition:*

- Darnell C. Watkins, “Rapid and Rigorous Qualitative Analysis: The “RADaR” Technique for Research Application.” *Journal for Qualitative Methods* (2017).
- Mark J. Belatto, “Analysis Methods for Qualitative Research.” *The Qualitative Report* (2018).
- Nora Kalpokaite, “Demystifying Qualitative Data Analysis for Qualitative Researchers: A Beginner’s Guide.” *The Qualitative Report* (2018).

*30% Women/All Fake Names Condition:*

- Darnell C. Watkins, “Rapid and Rigorous Qualitative Analysis: The “RADaR” Technique for Research Application.” *Journal for Qualitative Methods* (2017).
- Mark J. Belatto, “Analysis Methods for Qualitative Research.” *The Qualitative Report* (2018).
- Nora Kalpokaite, “Demystifying Qualitative Data Analysis for Qualitative Researchers: A Beginner’s Guide.” *The Qualitative Report* (2018).

Reading Memo: How does Kalpokaite’s approach to qualitative data analysis from Watkins’ RADaR method? Make sure you focus on how Watkins explains his methodology.

**Week 7**

**Field Experiments**

**Readings**

*10% Women/Mostly Real Names Condition:*

- Delia Baldassarri, “Field Experiments Across the Social Sciences.” *Annual Review of Sociology* (2017).
- Alan S. Gerber and Donald P. Green, “Field Experiments and Natural Experiments,” *Oxford Handbook of Political Science* (2011).
- Paolo Parigi, “Online Field Experiments.” *Social Psychology Quarterly* (2017).

*30% Women/Mostly Real Names Condition:*

- Delia Baldassarri, “Field Experiments Across the Social Sciences.” *Annual Review of Sociology* (2017).
- Alan S. Gerber and Donald P. Green, “Field Experiments and Natural Experiments,” *Oxford Handbook of Political Science* (2011).
- Paolo Parigi, “Online Field Experiments.” *Social Psychology Quarterly* (2017).

*10% Women/All Fake Names Condition:*

- Delilah Cessari, “Field Experiments in the Social Sciences: A Meta-Analysis.” *Annual Review of Sociology* (2017).
- Seth Gerber and Gerald P. Langley, “Field Experiments and Natural Experiments,” *Oxford Handbook of Political Science* (2011).
- Umberto Scipioni, “Conducting Field Experiments Online.” *Social Psychology Quarterly* (2017).

*30% Women/All Fake Names Condition:*

- Delilah Cessari, “Field Experiments in the Social Sciences: A Meta-Analysis.” *Annual Review of Sociology* (2017).
- Seth Gerber and Gerald P. Langley, “Field Experiments and Natural Experiments,” *Oxford Handbook of Political Science* (2011).
- Umberto Scipioni, “Conducting Field Experiments Online.” *Social Psychology Quarterly* (2017).

Reading Memo: What are the advantages and disadvantages of conducting online and field experiments in different contexts? Compare and contrast [Delia Baldassarri/Delilah Cessari] versus [Paolo Parigi’s/ Umberto Scipioni’s] approaches.

**Week 8**

**Regression Trees**

**Readings**

*10% Women/Mostly Real Names Condition:*

- Donald P. Green and Holger Kern, “Modeling Heterogeneous Treatment Effects in Survey Experiments with Bayesian Additive Regression Trees.” *Public Opinion Quarterly (*2012).
- Justin Grimmer, “ We Are All Social Scientists Now: How Big Data, Machine Learning, and Causal Inference Work Together.” *PS: Political Science & Politics* (2015).
- Nicolás L. Gutiérrez, Ray Hilborn & Omar Defeo, “Leadership, social capital and incentives promote successful fisheries.” *Nature* (2015).

*30% Women/Mostly Real Names Condition:*

- Donald P. Green and Holger Kern, “Modeling Heterogeneous Treatment Effects in Survey Experiments with Bayesian Additive Regression Trees.” *Public Opinion Quarterly* (2012).
- Christina Grimmer, “ We Are All Social Scientists Now: How Big Data, Machine Learning, and Causal Inference Work Together.” *PS: Political Science & Politics* (2015).
- Nicolás L. Gutiérrez, Ray Hilborn & Omar Defeo, “Leadership, social capital and incentives promote successful fisheries.” *Nature* (2015).

*10% Women/All Fake Names Condition:*

- Gerald P. Langley and Louis Nagel, “Heterogeneous Treatment Effects in Survey Experiments with Bayesian Additive Regression Trees: A Guide to Modeling.” *Public Opinion Quarterly* (2012).
- Justin Grimmer, “Big Data, Machine Learning, and Causal Inference Working Together.” *PS: Political Science & Politics* (2015).
- Nicolás L. Fernandez, George Mayer & Omar Caseo, “Leadership, social capital and incentives which can promote successful fisheries.” *Nature* (2015).

*30% Women/All Fake Names Condition:*

- Gerald P. Langley and Louis Nagel, “Heterogeneous Treatment Effects in Survey Experiments with Bayesian Additive Regression Trees: A Guide to Modeling.” *Public Opinion Quarterly* (2012).
- Mariana Grimmer, “Big Data, Machine Learning, and Causal Inference Working Together.” *PS: Political Science & Politics* (2015).
- Nicolás L. Fernandez, George Mayer & Omar Caseo, “Leadership, social capital and incentives which can promote successful fisheries.” *Nature* (2015).

Reading Memo: How do the [Nicolás Gutiérrez et al/Nicolás L. Gutiérrez et al] and the [Donald Green and Holger Kern/Gerald P. Langley and Louis Nagel] studies apply the concept that [Justin Grimmer/Christina Grimmer] talks about in [his/her] article?

**Week 9**

**Time Series Analysis**

**Readings**

*10% Women/Mostly Real Names Condition:*

- Beck, Nathaniel, Jonathan N. Katz, and Richard Tucker. “Time-series cross-section data." *American Political Science Review* 89.3 (1995): 634-647.
- Beck, Nathaniel, Jonathan N. Katz, and Richard Tucker. "Taking time seriously: Time-series-cross-section analysis with a binary dependent variable." *American Journal of Political Science* (1998): 1260-1288.
- John Laurence Thompson. “Deprivation and Political Violence in Northern Ireland, 1922-1985: A Time-Series Analysis” *Journal of Conflict Resolution* (1989).

*30% Women/Mostly Real Names Condition:*

- Beck, Nathaniel, Jonathan N. Katz, and Richard Tucker. “Time-series cross-section data." *American Political Science Review* 89.3 (1995): 634-647.
- Beck, Nathaniel, Jonathan N. Katz, and Richard Tucker. "Taking time seriously: Time-series-cross-section analysis with a binary dependent variable." A*merican Journal of Political Science* (1998): 1260-1288.
- Laura Thompson. “Deprivation and Political Violence in Northern Ireland, 1922-1985: A Time-Series Analysis” *Journal of Conflict Resolution* (1989).

*10% Women/All Fake Names Condition:*

- Barkley, Robert, Noris Satz, and Rich Taylor. “Time-series cross-section data." *Journal of Politics* (1995): 634-647.
- Barkley, Robert, Noris Satz, and Rich Taylor. "Exploring Time-series-cross-section analysis with a binary dependent variable." *American Journal of Political Science* (1998): 1260-1288.
- John L. Reily. “Political Violence in Northern Ireland, 1922-1995: A Time-Series Analysis” *Journal of Conflict Resolution* (1989).

*30% Women/All Fake Names Condition:*

- Barkley, Robert, Noris Salz, and Rich Taylor. “Time-series cross-section data." *Journal of Politics* 89.3 (1995): 634-647.
- Barkley, Robert, Noris Salz, and Rich Taylor. "Taking time seriously: exploring Time-series-cross-section analysis with a binary dependent variable." *American Journal of Political Science* (1998): 1260-1288.
- Laura L. Reily. “Political Violence in Northern Ireland, 1922-1995: A Time-Series Analysis” *Journal of Conflict Resolution* (1989).

Reading Memo: Please compare and contrast [Nathaniel Beck et al/Robert Barkley et al] and [John Thompson’s/Laura Thompson’s] approaches to time series analysis. Thompson’s article is a good example of applied methods, but pay attention to the way [he/she] explains the methods.

**Week 10**

**Writing and Publishing in the Social Sciences**

**Readings**

*10% Women/Mostly Real Names Condition:*

- John Gerring. ND. “General Advice on Social Science Writing.”
- David Almaveda. "How to Publish" PS: Political Science & Politics 39.1 (2006): 119-125.
- Patrick Dunleavy. *Authoring a PhD.* Chicago University Press, 2003.

*30% Women/Mostly Real Names Condition:*

- John Gerring. ND. “General Advice on Social Science Writing.”
- David Almaveda. "How to publish." PS: Political Science & Politics 39.1 (2006): 119-125.
- Patricia Dunleveth. *Authoring a PhD.* Chicago University Press, 2003.

*10% Women/All Fake Names Condition:*

- Jason Müller. ND. “Advice in General for Social Science Writing.”
- David Almaveda. "How to Publish" *PS: Political Science & Politics* 39.1 (2006): 119-125.
- Peter Dunleavy. *Authoring a Ph.D.* Chicago University Press, 2003.

*30% Women/All Fake Names Condition:*

- Jason Müller. ND. “Advice in General for Social Science Writing.”
- David Almaveda. "How to Publish" *PS: Political Science & Politics* 39.1 (2006): 119-125.
- Patricia Dunleveth. *Authoring a PhD*. Chicago University Press, 2003.

*** Your Final Paper is due to me by email on Tuesday of Week 10 ***
